# Volume Modulated Arc Therapy (VMAT) for pulmonary Stereotactic Body Radiotherapy (SBRT) in patients with lesions in close approximation to the chest wall

**DOI:** 10.3389/fonc.2013.00012

**Published:** 2013-02-22

**Authors:** Linda Ding, Yuan-Chyuan Lo, Sidney Kadish, David Goff, Richard S. Pieters, Geoffrey Graeber, Karl Uy, Syed Quadri, Richard Moser, Kevin Martin, John Day, Thomas J. FitzGerald

**Affiliations:** ^1^Department of Radiation Oncology, University of Massachusetts Medical School and the University of Massachusetts Memorial Health Care SystemWorcester, MA, USA; ^2^Department of Thoracic Surgery, University of Massachusetts Medical School and the University of Massachusetts Memorial Health Care SystemWorcester, MA, USA; ^3^Department of Neurosurgery, University of Massachusetts Medical School and the University of Massachusetts Memorial Health Care SystemWorcester, MA, USA; ^4^Department of Medicine, University of Massachusetts Medical School and the University of Massachusetts Memorial Health Care SystemWorcester, MA, USA; ^5^Department of Medicine, St. Vincent HospitalWorcester, MA, USA; ^6^Department of Medicine, Day Kimball HospitalPutnam, CT, USA

**Keywords:** Volume Modulated Arc Therapy, radiation therapy, intrathoracic lesions, stereotactic body radiotherapy, chest wall

## Abstract

**Purpose:** Chest wall pain and discomfort has been recognized as a significant late effect of radiation therapy in historical and modern treatment models. Stereotactic Body Radiotherapy (SBRT) is becoming an important treatment tool in oncology care for patients with intrathoracic lesions. For lesions in close approximation to the chest wall with motion management, SBRT techniques can deliver high dose to the chest wall. As an unintended target of consequence, there is possibility of imposing significant chest wall pain and discomfort as a late effect of therapy. The purpose of this paper is to evaluate the potential role of Volume Modulated Arc Therapy (VMAT) technologies in decreasing chest wall dose in SBRT treatment of pulmonary lesions in close approximation to the chest wall.

**Materials and Methods:** Ten patients with pulmonary lesions of various sizes and tomography in close approximation to the chest wall were selected for retrospective review. All volumes including tumor target, chest wall, ribs, and lung were contoured with maximal intensity projection maps and four-dimensional computer tomography planning. Radiation therapy planning consisted of static techniques including Intensity Modulated Radiation Therapy compared to VMAT therapy to a dose of 60 Gy in 12 Gy fraction dose. Dose volume histogram to rib, chest wall, and lung were compared between plans with statistical analysis.

**Results:** In all patients, dose and volume were improved to ribs and chest wall using VMAT technologies compared to static field techniques. On average, volume receiving 30 Gy to the chest wall was improved by 74%; the ribs by 60%. In only one patient did the VMAT treatment technique increase pulmonary volume receiving 20 Gy (V20).

**Conclusions:** VMAT technology has potential of limiting radiation dose to sensitive chest wall regions in patients with lesions in close approximation to this structure. This would also have potential value to lesions treated with SBRT in other body regions where targets abut critical structures.

## Introduction

Radiation therapy is an important treatment modality in cancer care. Acute normal tissue effects during therapy generally affect tissues of rapid self renewal potential including skin and mucosal surfaces. Late effects from treatment can affect tissues of both rapid and limited self renewal potential. Injuries to tissues of limited self renewal potential including bone, muscle, and nerve often become chronic in nature and result in significant pain with limited successful treatment options. In early breast cancer management, patients were treated with enface photon techniques with varied fractionation strategies including strategies not commonly employed in modern care. On occasion, these patients would develop chest wall injury and rib fracture (Dalinka et al., 1974; Overgaard, 1988). Even with more modern pre-IMRT breast cancer therapy techniques, areas of radiation dose inhomogeneity were identified in medial and lateral rib/chest wall/brachial plexus segments with the level of dose inhomogeneity exceeding 115% of prescription in significant volumes of the breast tissue and chest wall. Bone scans obtained at various time points from therapy would reveal activity in these locations and anecdotal rib fractures and chest wall soft tissue injury were seen in this population of patients. Intensity modulation has permitted improvement in radiation dose distribution in this population. Improvements in treatment technology including modern image guidance have re-established the role of accelerated treatment fractionation schedules in selected patients to strategic targets including lesions requiring motion management. Stereotactic radiosurgery (SRS) and radiation therapy have become important tools for modern care for the oncology patients. Both primary lesions and metastatic lesions in multiple body areas are successfully treated with radiosurgery and stereotactic radiation therapy. High fraction therapy to limited volumes may have a separate successful mechanism of tumor cell killing influencing the use of this form of therapy. High dose limited fraction radiation therapy used in SRS and SBRT is uniquely challenging when tumor target volumes lie in juxtaposition to normal tissue structures of limited self renewal potential such as chest wall, rib, and nerve. In this situation the risk of long-term injury is more likely. Chest wall injury and pain syndrome can be a significant and debilitating effect of therapy with limited treatment options once the injury becomes clinically apparent. There is increasing evidence that the risk of chest wall injury is related to the volume of chest wall receiving high dose radiation as an unintended target. Mutter and colleagues have recently demonstrated a significant increase in chest wall injury and pain syndrome driven by the volume of chest wall receiving 30 Gy (V30) (Mutter et al., 2012). Other investigators have demonstrated similar findings in retrospective review of radiosurgery treatment plans including issues associated with body habitus influencing treatment outcome (Voroney et al., 2009; Dunlap et al., 2010; Welsh et al., 2010).

In this paper, we evaluate the role of Volumetric Modulated Arc Therapy (VMAT) in limiting radiation dose to the chest wall volume in patients with pulmonary lesions in close approximation to the chest wall. The objective is to determine if this planning strategy may result in quantitative improvement of radiation dose to the chest wall in this important clinical situation.

## Materials and methods

Ten patients with pulmonary lesions in close approximation to the chest wall (average 2 cm) were chosen in retrospect from our patient population for this review as these patients appeared suitable for this analysis. Eight patients had primary disease of the lung (Stage 1, non-small cell lung cancer-NSCL) and two patients had metastatic disease with an average age at the time of treatment of 71 years (range 52–91). As part of the simulation process, all patients signed consent to permit use of their imaging and radiation therapy planning objects for education and research in a de-identified format.

### Motion management

All patients underwent free breathing 4D computer tomography radiation therapy planning. For patients with motion less than 15 mm, the clinical target volume (CTV) from the maximal intensity projection plus motion was chosen as the internal target volume (ITV). For patients with motion greater than 15 mm, patients were planned with amplitude breathing defined and reproduced with cone beam CT validation of the target immediately prior to treatment execution. Planning target volume (PTV) was 2 mm for both planning strategies. Each patient was fitted with a Vac-loc device (Culver City, IA) fitted with a wingboard for radiation therapy planning and treatment.

### Radiation therapy planning

For static field planning, patients were planned using 50% IMRT beam strategy and 50% static field. On average, seven fields were used for treatment plans using static fields. For VMAT planning, two one-half arcs were used and directed to the same target volumes.

Optimization constraints included 40 Gy to less than 10% of the chest wall/ribs and 20 Gy to less than 20% of the affected lung volume or better. The constraints were identical for both planning strategies.

### Radiation therapy dose

All patients were planned to receive 60 Gy in 12 Gy fractions used by both treatment plans for purpose of comparison.

### Normal tissue structures

The chest wall was drawn from the inner surface of the rib and included 2 cm beyond this point from the sternum to the facet joint of the vertebral body. The rib was drawn as a separate target by using 4D CT in maximum intensity projection. The lung was drawn as a separate target. The V20 was calculated relative to the entire lung volume in the affected lung and compared between each plan. The V30 of the chest wall and rib was compared between the two plans for each patient.

### Statistics

Student *T*-test was used to compare difference in V30 for the rib and chest wall and the pulmonary V20 between both plans for each patient.

## Results

Patient demographics, CTV/PTV volumes, and distance of the target from the chest wall are listed in Table 1. The results for the V30 to the chest wall and ribs as well as the V20 to the pulmonary parenchyma are demonstrated in Table 2. The absolute volumes of rib, chest wall, and pulmonary parenchyma are listed in Table 3a,b,c, respectively. Individual patient maximum tumor motion is listed in Table 4. All patients demonstrated improvement in V30 for the chest wall and rib using VMAT planning. The average improvement to the chest wall was 74.3% and to the ribs 60.8% using the VMAT plan (*p* < 0.05). Only one patient demonstrated an increase in pulmonary V20 with the VMAT plan.

**Table 1:** **Patient demographics and tumor statistics**.

**Patient (age during treatment time)**	**CTV(cc)**	**PTV(cc)**	**Tumor type**	**Distance to the closest rib (cm)**
1 (91)	38	74.3	NSCL	0.8
2 (77)	17.9	43.3	Metastasis/breast	2.2
3 (80)	16.1	49.8	NSCL	1.5
4 (74)	14.3	74.8	NSCL	2.5
5 (62)	11.4	35.1	NSCL	2.4
6 (64)	1.7	14.7	NSLC	2.8
7 (62)	0.9	9	Metastasis/colon	1.0
8 (52)	9.2	38.5	NSCL	1.8
9 (84)	25.6	47	NSCL	2.0
10 (61)	4.7	18	NSCL	2.3
Average (71 years old)	14.0	40.4		1.9

**Table 2:** **V30 for chest wall and ribs was reduced for all 10 patients from original planning technique**.

**Patient**	**Chest wall volume receive >30 Gy (%)**	**Ribs receive >30 Gy (%)**	**V20 lung dose (%)**
1	−65.5	−59.2	−2.4
2	−60.5	−91.1	−32.0
3	−32.2	−11.1	−6.5
4	−93.3	−51.4	12.6
5	−55.0	−47.9	−18.3
6	−57.0	−100.0	−54.1
7	−100.0	−69.1	−26.3
8	−62.1	−37.1	−3.9
9	−54.1	−40.9	−16.7
10	−55.6	−100.0	−52.0

V20 lung dose decreased for 9 of the 10 patients. The data is presented as the percent decrease in volume treated using VMAT technology.

**Table 3a:** **Rib volume receiving 30 Gy**.

**Patient no.**	**Hybrid SBRT (cc)**	**RA SBRT (cc)**
1	38.3	15.6
2	5.6	0.5
3	10.2	9.0
4	17.3	8.4
5	19.9	10.4
6	3.1	0.0
7	3.3	1.0
8	15.1	9.5
9	14.1	8.4
10	2.7	0
Average	13.0	6.3

**Table 3b:** **Chest wall (2 cm) volume receiving 30 Gy**.

**Patient no.**	**Hybrid IMRT SBRT (cc)**	**RA SBRT (cc)**
1	501.6	139.7
2	30	3.1
3	134.1	65.7
4	347	56.3
5	330.6	56
6	56.7	0
7	17.1	7.7
8	247.7	55.8
9	219.2	72
10	55.4	20
Average	193.9	47.6

**Table 3c:** **Percent lung volume receiving 20 Gy**.

**Patient no.**	**Hybrid IMRT SBRT (%)**	**RA SBRT (%)**
1	12.3	12.0
2	22.5	15.3
3	13.9	13.0
4	8.0	6.5
5	3.8	2.8
6	17.6	19.8
7	9.8	4.5
8	15.4	14.8
9	8.4	7.0
10	12.3	5.9
Average	12.4	10.2

**Table 4:** **Maximum tumor motion measured on 4D CT**.

**Patient no.**	**1**	**2**	**3**	**4**	**5**	**6**	**7**	**8**	**9**	**10**
Motion (mm)	8.5	9	5	3	9.4	12.4	10.8	9.8	2	3

## Discussion

Pain and discomfort of the chest wall can be a late effect of radiation management and treatment of this effect can be unsatisfactory for both the patient and the physician. Often treatment management options are limited to analgesia and nerve blocks, each of which is often partially effective.

Traditional radiation therapy fractionation regimens can be associated with the development of discomfort in the chest wall and in more historical models rib fractures were identified on occasion as part of breast cancer management (Dalinka et al., 1974; Overgaard, 1988; Pierce et al., 1992; Hall, 2000; Jackson et al., 2010). More modern radiation therapy treatment planning strategies including volumetric treatment planning with intensity modulation radiation therapy treatment execution permit thorough examination of the radiation therapy dose to chest wall structures, often permitting planning strategies to limit the volume and extent of radiation dose inhomogeneity with the chest wall target. These advances, including the use of integrated advanced technology imaging techniques with real time target validation, have permitted the field of radiation oncology to re-visit the use of high dose limited fractionation treatment schedules including stereotactic therapy for multiple body regions and tumor targets. For tumor targets in close approximation to the chest wall, investigators are recognizing there is risk of injury to the chest wall with accelerated fraction radiation therapy, especially when high volumes of the chest wall receive greater than 30 Gy. One series suggests that the risk of injury to the chest wall with radiosurgery techniques is greater than 40% when lesions are within 2 cm of the chest wall (Asai et al., 2012). Therefore, when the tumor target is in close approximation to the chest wall, there would be a higher risk using more traditional radiotherapy technologies that use static fields as there would be more rib and chest wall volume in the therapy field (Dalinka et al., 1974; Overgaard, 1988; Pierce et al., 1992; Meric et al., 2002; Shioyama et al., 2005; Zimmerman et al., 2006; Kyas et al., 2007; Baumann et al., 2009; Petersson et al., 2009; Siva et al., 2010; Andolino et al., 2011; Nambu et al., 2011; Stephans et al., 2012). This risk maybe further exaggerated with larger PTVs needed for set up uncertainty indicating the importance of immobilization and treatment reproducibility. In our experience, we have found that personalized Vac-Loc with integrated wingboard works well for daily reproducibility of patient set up coupled with shallow breathing techniques. We have found that multiple compression devices have introduced additional error into the patient set up and have not demonstrated an advantage in patient care or reproducibility of each treatment. We identify the breathing cycle amplitude during the simulation process and have successfully reproduced the target with cone beam CT validation by re-establishing the breathing amplitude cycle used at the time of simulation. In patients with significant motion or change in respiratory status, we perform a second simulation to validate that the ITV is identical to the volume established at the time of primary simulation. To date, no patient has sustained a chest wall injury and none have relapsed in the local target volume.

**Figure 1:**
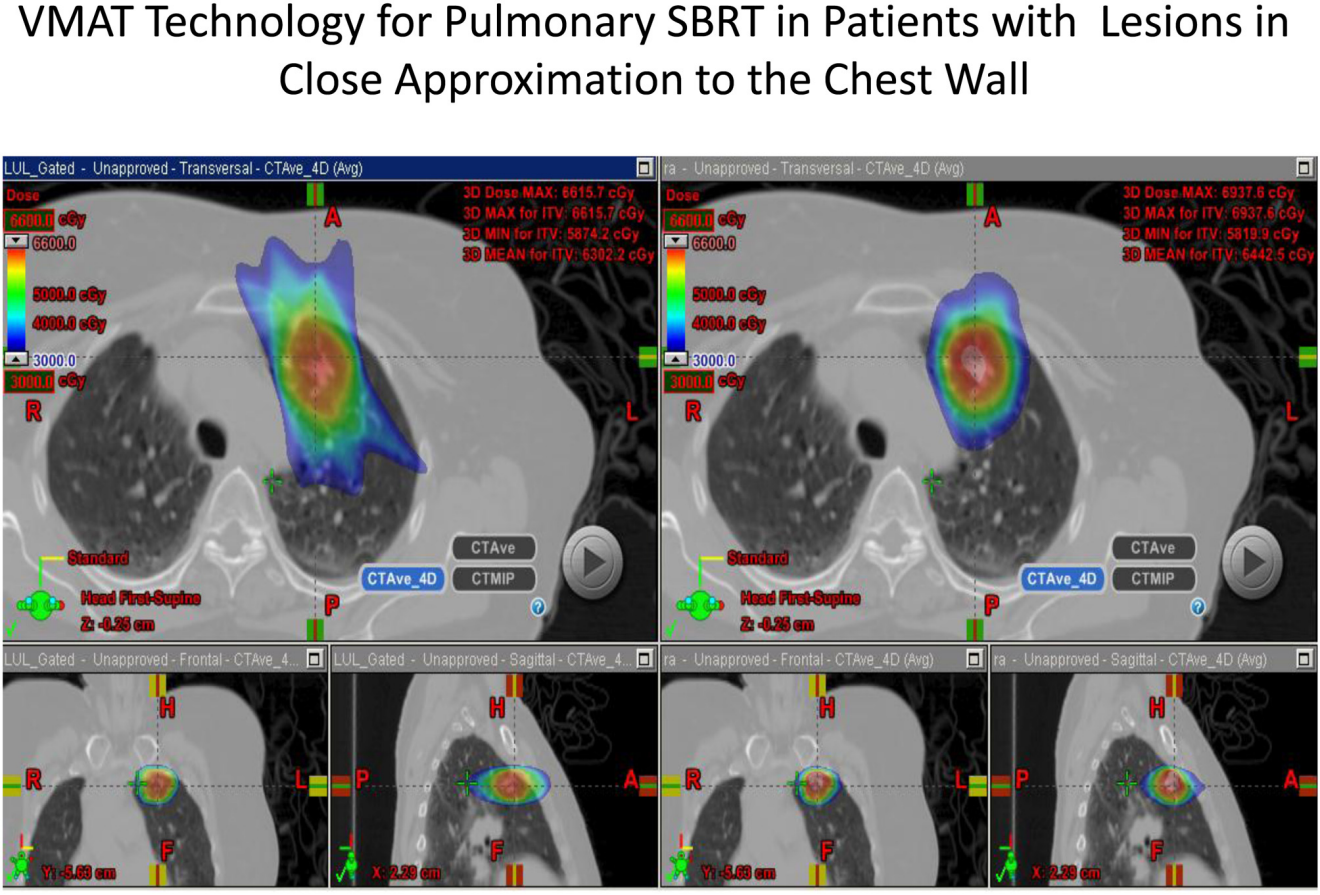
**Demonstrates improvement in dosimetry in a lesion in the anterior segment of the left upper lobe with the static field plan on the left and the VMAT plan on the right**.

**Figure 2:**
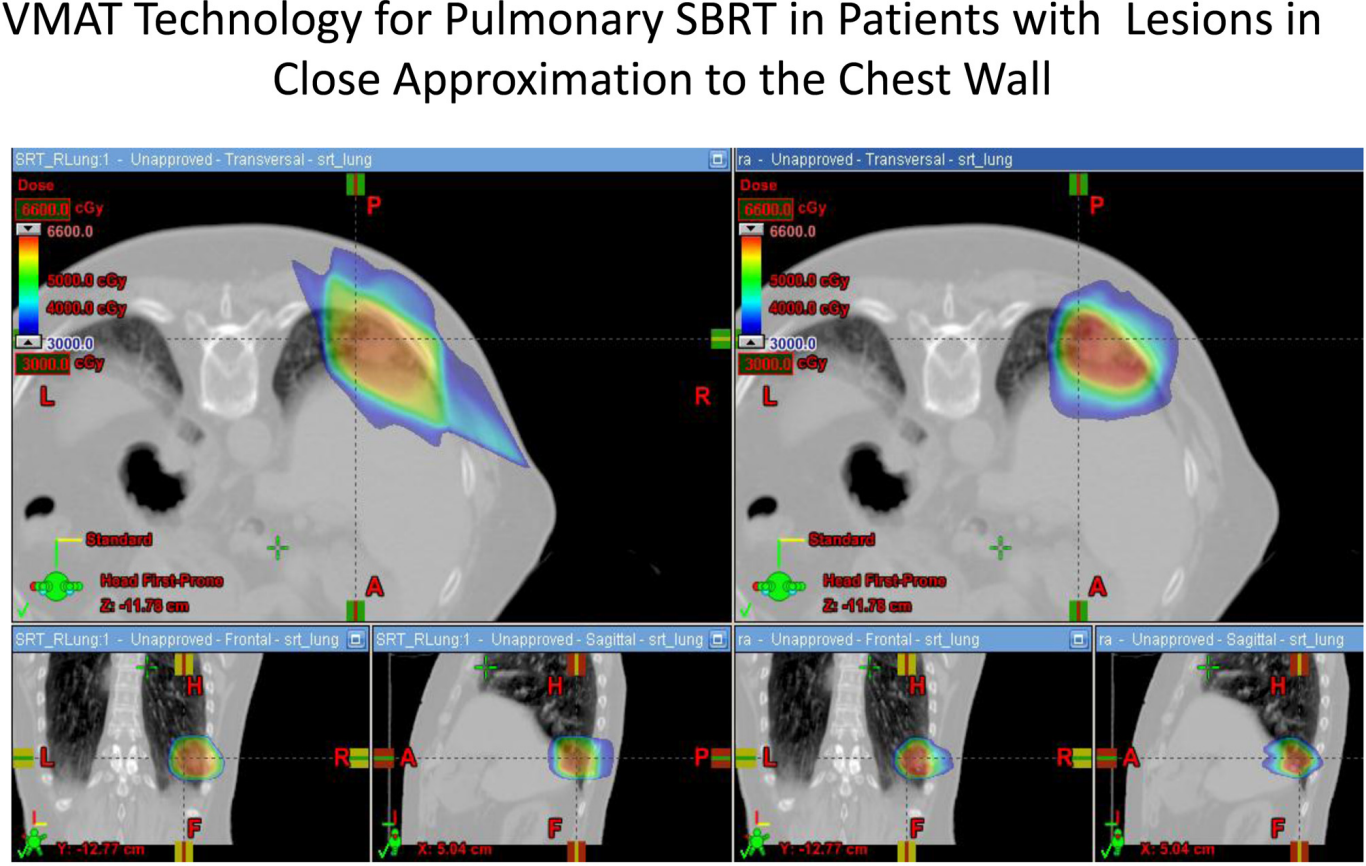
**Demonstrates improvement in dosimetry to the chest wall and ribs in a patient in the prone position with a lesion in the posterior segment of the right lower lobe.** The static field plan is on the left and the VMAT plan is on the right.

The use of modulated arc radiotherapy appears in our study to have significant potential benefit for patient care in the situation where tumor comes in close approximation to the chest wall region. VMAT can accommodate the multiple sloping surfaces of the chest wall and the dynamic nature of the simultaneous dual motion of both the gantry and multileaf collimators permits potentially more optimal radiation therapy treatment planning and therapy execution. VMAT technology may decrease the risk of injury to this selected patient population. In our study, significant decreases in radiation dose to both chest wall and rib targets were seen in side by side comparison between VMAT technology and traditional SBRT treatment plans with static fields. Figures 1 and 2 demonstrate the improvement achievable in supine and prone positions, respectively. In our analysis the average number of fields was seven (7) for non-VMAT planning. Another advantage to VMAT therapy is speed of treatment delivery and subsequent improvement in therapy dose rate. Traditional static field radiosurgery treatment techniques with multiple fields can take a significant amount of time to complete, often greater than 30 min coupled with time for image validation. VMAT therapy can take place in a much more abbreviated time frame (a few min) further decreasing risk of target motion during therapy as well as provide a more comfortable treatment environment for the patient. The primary concern of arc therapy is in this population that arc treatment will increase dose to pulmonary parenchyma in patients treated with this technique. We note only one patient experienced an increase in pulmonary V20 with optimal VMAT planning relative to planning with static fields (patient 4/12% increase). It is likely VMAT technology can play an important role in hepatic radiotherapy when tumor comes in close approximation to the chest wall as well as other tumor targets in multiple body locations that abut critical normal tissue structures.

### Conflict of interest statement

The authors declare that the research was conducted in the absence of any commercial or financial relationships that could be construed as a potential conflict of interest.
